# Cryopreservation of Embryos of the Mediterranean Fruit Fly *Ceratitis capitata* Vienna 8 Genetic Sexing Strain

**DOI:** 10.1371/journal.pone.0160232

**Published:** 2016-08-18

**Authors:** Antonios A. Augustinos, Arun Rajamohan, Georgios A. Kyritsis, Antigone Zacharopoulou, Ihsan ul Haq, Asya Targovska, Carlos Caceres, Kostas Bourtzis, Adly M. M. Abd-Alla

**Affiliations:** 1 Insect Pest Control Laboratory, Joint FAO/IAEA Division of Nuclear Techniques in Food and Agriculture, Vienna, Austria; 2 Insect Genetics and Biochemistry Unit, Biosciences Research Lab, USDA, Fargo, ND, 58105, United States of America; 3 Department of Biology, University of Patras, Patras, Greece; Virginia Tech, UNITED STATES

## Abstract

The Mediterranean fruit fly, *Ceratitis capitata*, is one of the most serious pests of fruit crops world-wide. During the last decades, area-wide pest management (AW-IPM) approaches with a sterile insect technique (SIT) component have been used to control populations of this pest in an effective and environment-friendly manner. The development of genetic sexing strains (GSS), such as the Vienna 8 strain, has been played a major role in increasing the efficacy and reducing the cost of SIT programs. However, mass rearing, extensive inbreeding, possible bottleneck phenomena and hitch-hiking effects might pose major risks for deterioration and loss of important genetic characteristics of domesticated insect. In the present study, we present a modified procedure to cryopreserve the embryos of the medfly Vienna 8 GSS based on vitrification and used this strain as insect model to assess the impact of the cryopreservation process on the genetic structure of the cryopreserved insects. Forty-eight hours old embryos, incubated at 24°C, were found to be the most suitable developmental stage for cryopreservation treatment for high production of acceptable hatch rate (38%). Our data suggest the absence of any negative impact of the cryopreservation process on egg hatch rate, pupation rates, adult emergence rates and stability of the temperature sensitive lethal (*tsl)* character on two established cryopreserved lines (flies emerged from cryopreserved embryos), named V8-118 and V8-228. Taken together, our study provides an optimized procedure to cryopreserve the medfly Vienna 8 GSS and documents the absence of any negative impact on the genetic structure and quality of the strain. Benefits and sceneries for utilization of this technology to support operational SIT projects are discussed in this paper.

## Introduction

The Mediterranean fruit fly or medfly, *Ceratitis capitata*, is one of the most serious pests of fruit crops world-wide causing direct damage to a wide range of high value fruit and vegetable crops thus leading to significant yield reductions and loss of quality [1, 2]. In addition, quarantine restrictions imposed by medfly-free countries impact horticultural exports from countries where the pest is present [3]. The control programs of this serious pest involve the use of pesticide sprays, poisoned baits and area-wide integrated pest management AW-IPM) approaches with a sterile insect technique (SIT) component. SIT involves mass rearing large numbers of flies, which are sexually sterilized by exposing to irradiation and release to reproductively compete with the wild population [4–6].

There have been several successful SIT projects which were based on the release of both sterile males and females as was the case of the eradication program against the New World screwworm *Cochliomyia hominivorax* (Coquerel) [7, 8]. However, it has been shown that in SIT projects the bisexual releases are far less effective than male-only releases [9–13]. This finding resulted in the development and large scale use of genetic sexing strains (GSS) using classical genetic approaches including the induced chromosome rearrangements and isolation of specific mutations [14, 15]. GSS are based on the principle of the sex-specific pseudo-linkage of a selectable marker [15]. Although GSS have been developed for several species [16–18], perhaps the most successful ones in the history of large scale operational SIT programs have been the medfly Vienna GSS which were based on the use of pupal color and the presence of temperature-sensitive lethal (*tsl*) genes as selectable markers [15, 19–22].

Different GSS have been constructed for the Mediterranean fruit fly, using classical genetic approaches, and optimized through years to reduce the cost and increase the effectiveness of AW-IPM [15, 23–25]. The ones currently used in SIT programs worldwide are the Vienna 7 and, mainly, the Vienna 8 strain. Both strains (Vienna 7 and Vienna 8) contains (i) the temperature sensitive lethal (*tsl*) gene and the white pupae (wp) selectable marker on autosome 5, along with a T(Y-5) autosome translocation which pseudolinks the wild-type allele of the aforementioned selectable markers to the male sex. In this strains, the incubation of 24 h old eggs at 34°C for 12–24 hours eliminates all females (homozygous for *tsl*), leading therefore to the production and release of males only. However, both the rearing and release process may have a direct impact on reared males’ quality, reducing their capability to compete with wild males for mating with wild females in the field [1]. In some species, males reared on a large scale become less competitive over generations of rearing in the laboratory [26–28]. Recent studies have clearly shown that lab domestication and mass rearing may critically affect the competitiveness of mass-reared insects thus resulting in to the loss of the initial biological properties of the insect strain [29–31]. In such cases where domestication adversely affects the quality traits, cryopreservation technologies could help overcoming the abovementioned constraints and help to maintain the initial characteristics of the insect strain.

Cryopreservation is the process of preserving cells or whole tissues (embryos or sperm) or any other substances susceptible to damage caused by chemical reactivity or time by optimally dehydrating them using a partial pressure gradient and storing them at very low temperatures (viz liquid nitrogen at -196°C). The cryopreservation technology has previously been successfully implemented in a few insects such as the New World Screwworm *Cochliomyia hominivorax* [32], the sheep blow fly, *Lucilia cuprina* [33, 34], the housefly *Musca domestica* [35, 36], the Mexican fruit fly, *Anastrepha ludens* [37], the Caribbean fruit fly, *Anastrepha suspensa* [35, 37–39] and in medflies as well [38]. None of the previous studies assessed the impact of the cryopreservation process on the genetic structure of the preserved insects.

In this paper we describe a modify cryopreservation protocol for medfly Genetic sexing strain Vienna 8 GSS based on the presence of temperature-sensitive lethal (*tsl*) genes and use this strain as insect model to assess the impact of the cryopreservation process on the quality and genetic structure of this particular strain which is being used in large scale SIT operational programs worldwide.

## Materials and Methods

### Cryopreservation

**Fig 1:**
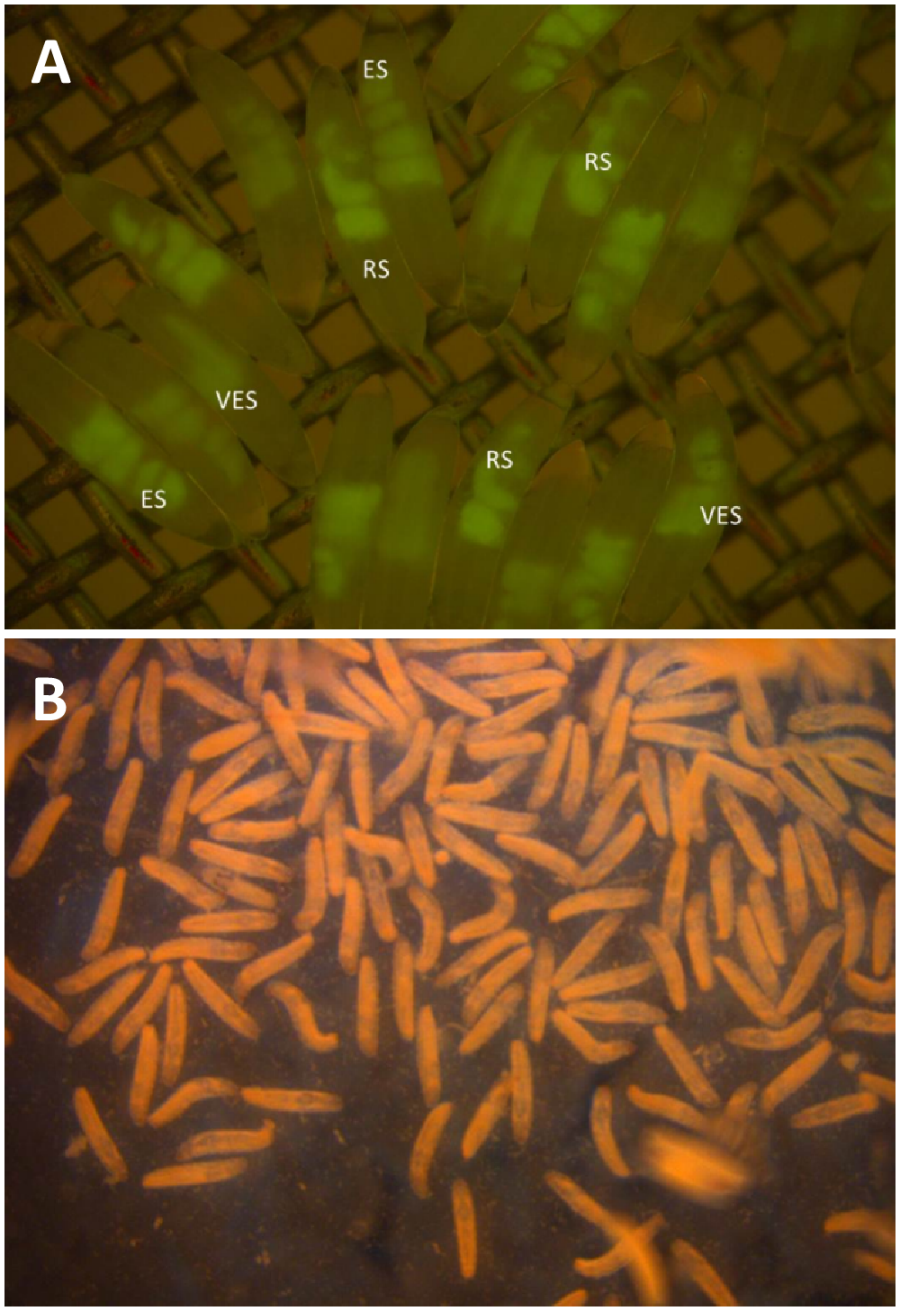
Embryonic stages and larvae sorted from cryopreserved embryos of Vienna 8. (A) Suitable embryonic stage of Vienna-8 for starting the cryopreservation process characterized by the helical shape of the embryos gut with minimum amount of yolk. Embryos were observed with Leica M205 FA Stereomicroscope in phase contrast under UV light and GFP filter. (RS: right stage). (B) Vienna-8 larvae sorted from cryopreserved embryos.

Freshly laid eggs (embryos) of a mass reared medfly Vienna 8 GSS were collected for a period of 30–60 minutes at the Joint FAO/IAEA Insect Pest Control Laboratory. Around 168.000 adult flies were maintained in 200 x 200 x 20 cm cages as previously described by Vargas [40] and fed on adult diet consisting of sugar and yeast in a 3:1 ratio by weight. The flies were maintained at 23–24°C and 60–68% relative humidity. The embryos were spread evenly on a moist filter paper in a Petri plate and incubated at 24°C until they were developmentally ready for cryopreservation (48 ± 0.5 hours). Cues such as the helical shape of the embryos gut (Fig 1) and the amount of yolk in the gut were used to determine the appropriate developmental stage for cryopreservation [38]. The embryos were processed for cryopreservation using two methods that involve manual method or using a home-made robot and transfer of the embryos to the liquid nitrogen as described in [38]. The procedure consisted of a permeabilization step followed by cryoprotectant loading and subsequent dehydration before exposure to the cryogen to affect the process of vitrification. The permeabilization procedure includes two steps—embryonic dechorionation followed by the removal of the surface lipids on the vitelline membrane. After permeabilization, the embryo was pretreated with a permeating cryoprotectant and then dehydrated in a cocktail of permeating cryoprotectant and a non-permeating sugar solution.

### Dechorionation and permeabilization

While the protocols for dechorionation and permeabilization were similar to the procedure detailed in Rajamohan et al. [38], the number of embryos and exposure timing were optimized for the medfly Vienna 8 strain used in this study. Approximately 150–200 embryos were placed in a 4 cm diameter 150 μm mesh basket. The embryos were immersed and agitated in a 25% freshly prepared solution of sodium hypochlorite (Concentrated Clorox^®^, Splash-Less^™^ Regular Bleach) and repeatedly swirled in the solution and pulled out (≤ 2 min) until most of the embryos were observed to float on the surface. Thereafter the embryos were quickly washed in running tap water for 3 min until no odour of sodium hypochlorite could be noted. The embryos and the inner walls of the mesh basket were bloated semi-dry with a tissue paper. The developmental stages of the embryos was assessed using Leica M205 FA Stereomicroscope in phase-contrast under incident UV light and GFP filter while the embryos were floating on Schneider’s insect cell culture medium (Sigma-Aldrich, MO, USA). Embryos were counted and scored to determine the number of embryos in ‘stage proper’ as described in Rajamohan et al. [38]. If the embryos were noticed to not have reached the requisite developmental stage, incubation was continued at room temperature with the embryos left floating on the Schneider’s medium.

After removing excess water from the mesh basket containing the embryos, they were rinsed in 2-propanol (99.9% & <0.001% water; Sigma-Aldrich; MO, USA) for a period not exceeding 20 seconds to completely de-wet the embryos. The embryos were dried quickly by blotting excess 2-propanol and using a stream of air for at least 1 minute. The embryos were then rinsed in hexanes (99.5%; Sigma-Aldrich / Burdick Johnson, USA) for exactly 40 seconds and continuously shaken to avoid clumping of the embryos. The embryos were again blotted and air dried for a minute and floated on Schneider’s medium.

### Cryoprotectant loading, cryopreservation and embryo resuscitation

The permeabilized embryos were cryoprotected, dehydrated and vitrified as described in Rajamohan and his colleagues. [38]. In brief, the embryos were transferred to a cocktail of 1.8 M 1,2-ethanediol (Reagent grade; Sigma-Aldrich, MO, USA) diluted in Schneider’s insect cell culture medium and incubated at room temperature for 20 minutes. This cryoprotectant pretreatment was done in a mesh basket described in the permeabilization step that was placed partially immersed in the solution. The basket containing the embryos was removed and excess solution was blotted by placing it on a tissue paper. The embryos were then transferred to an ice-cold solution of Schneider’s insect cell culture medium containing 7.0 M 1,2-ethanediol, 0.5M trehalose dehydrate (Swanson Health Foods, Ltd., Fargo, ND, USA) and 5% polyethylene glycol (average molecular weight 8000; Sigma-Aldrich, MO, USA). This solution is hereafter referred to as the ‘vitrification solution’ wherein the embryos were incubated over ice for a period not exceeding 15 minutes.

The embryos were vitrified precisely as previously described in [38]. Prior to the cryogen exposure the embryos were transferred to a polycarbonate membrane (0.8 um; Nuclepore^™^ track-etch membrane, GE Healthcare Life Science, PA, USA) and the excess of vitrification solution was removed by blotting the membrane over a filter paper for less than 15 seconds. Thereafter the membrane was plunged into liquefied nitrogen after a one minute exposure to the vapor nitrogen by suspending the membrane at approximately 1 cm above the liquefied nitrogen.

To resuscitate the cryopreserved embryos, the membrane was resuspended in the vapor nitrogen at not more than 1 cm above the liquid nitrogen and held there until the membrane dried completely. Thereafter the membrane was immersed rapidly into a 0.5 M trehalose solution with the embryo side for the membrane facing the solution. The membrane was shaken to dislodge the embryos into the solution and after 2 minutes the solution was replaced with Schneider’s medium. This medium was replaced thrice every 10 minutes. After three such replacements, the embryos were permitted to develop in the medium in an incubator at 24°C. After 24 hours, the proportion of embryos that had hatched was recorded and the hatched larvae were picked from the solution using a plastic pipette and placed on the larval diet as described by Tanaka et al. [41] to permit larval growth and pupation. Emerged flies were maintained in small cages to establish two cryopreserved lines (V8-118 developed by manual processing and V8-228 by processing with Robot) of the medfly Vienna 8 GSS. The adults of these lines were inbred, scaled up and used for the quality control tests. The entire cryopreservation process was conducted in aseptic conditions under a laminar flow to avoid bacterial contamination.

### Quality control (QC) of the recovered lines

The following parameters of the Vienna 8 GSS were assessed: a) recovery rates, as described by the percentages of egg hatch, pupation and adult emergence in different temperatures, b) recombination rates, as described by the percentage of unexpected adults (males emerging from white pupae and females emerging from brown pupae) and c) percentages of ‘escapers’ (white pupae and female flies surviving the heat shock treatment).

The need for increased numbers of synchronized eggs did not allow performing the Quality Control before F2 generation. Therefore, for recovery and recombination rates, two different screenings were performed, based on tests described previously [15, 16]: i) Forty ml pupae screening: 40 ml pupae (pupae number is measured volumetrically) were collected from each strain, in the 2^nd^ generation after revival from cryopreservation. White and brown pupae were separated and were allowed to emerge. After emergence, the number of males and females was counted. For comparative reasons and in order to avoid deviations deriving from technical handling, the medfly Vienna 8 GSS from which the cryopreserved lines originated was also included in the analysis (Table 1), ii) *tsl* screening and recovery analysis: females from the 2^nd^ generation after revival from cryopreservation were used. Briefly, 1800 eggs (5 h morning collections, 8.00–13.00) per day were collected for three consecutive days, from 4–8 days old females. Eggs were placed on black strips (100/strip) in petri dishes with carrot diet. A total of 18 strips were prepared per day and left at 25°C for 24 h. After 24 h, eggs were placed in five different temperatures (25, 31, 32, 33, 34 and 35°C). Three replicas were placed in each temperature. After 24 h, eggs were placed again in 25°C. In Day 5, egg hatch was counted. After pupation, brown and white pupae were counted and kept separate. After emergence, the number of adults (males and females separately) was counted. For comparative reasons and in order to avoid deviations deriving from technical handling, the medfly Vienna 8 GSS from which the cryopreserved lines originated was also included in the analysis (Table 2). Averages, standard errors and single factor ANOVA were calculated in Excel, utilizing the Analysis ToolPak add-in. A number of indices were calculated, such as egg hatch, pupation (total, white pupae and brown pupae), adult emergence (total, males and females) and sex ratio (in pupal and adult stages). Prior to ANOVA, Levene’s test was performed, to assess the homogeneity of the variances at the significant level of 0.05. The null hypothesis of homogeneity was not rejected for any of the measurements, allowing thus to proceed with single factor ANOVA. Levene’s test was also performed in Excel, using the Analysis ToolPak add-in.

**Table 1:** Recovery rates from the 40 ml of pupae and 5400 eggs tests. Note that, only the data for the 900 of the 5400 eggs incubated at 25°C are included in the table. Sex ratio is also indicated.

		40 ml pupae test			5400 eggs test		
		V8-118	V8-228	V8-F40	V8-118	V8-228	V8_F40
**Males**	Brown pupae	1057	1265	1051	242	231	244
	White pupae	0	0	0	0	0	0
**Females**	Brown pupae	0	0	0	0	0	0
	White pupae	825	743	996	221	172	209
**Total emergence** (males and females)		1882	2008	2047	463	403	453
**Sex ratio** (males/females)		1.28	1.70	1.24	1.08	1.33	1.15

**Table 2:** Summary of the protocol used for the *tsl* screening and Quality Control analysis of the medfly Vienna 8 GSS.

	1^st^ egg collection	2^nd^ egg collection	3^rd^ egg collection
Day 1	18x100 eggs		
Day2	transfer to incubators	18x100 eggs	
Day3	Return to 25°C	transfer to incubators	18x100 eggs
Day4		Return to 25°C	transfer to incubators
Day5	Count egg hatch		Return to 25°C
Day6		Count egg hatch	
Day7			Count egg hatch
Day X	Count pupae, separate brown-white		
Day Y	Count adult emergence, plus males-females	Count pupae, separate brown-white	
		Count adult emergence, plus males-females	Count pupae, separate brown-white
			Count adult emergence, plus males-females

### Cytogenetic characterization

Polytene chromosome squashes were prepared from 5–6 days old male pupae, through the isolation of trichogen cells of the spatulate superior orbital bristles [42, 43]. This technique has been used for the characterization of the Y—autosome translocations involved in the development of medfly GSSs [15, 44]. At least 10 nuclei were analyzed per strain, representing at least 10 individual flies. Due to the reduced percentage of hatch and the need to scale up the colonies, cytogenetic characterization was performed after the 2^nd^ generation from the revival.

### Mitochondrial haplotypes characterization

The PCR-RFLP test performed has been developed to differentiate among different medfly Vienna GSSs and also among different natural populations [45, 46]. DNA was extracted from 10 individual flies per strain, using the DNeasy Blood and Tissue kit (Qiagen). Four different mitochondrial regions were PCR amplified using the primer pairs described in Table 3. PCRs were carried out in a total volume of 30 μl, using 15 μl of the 2X Taq PCR master Mix (Qiagen), 1.5 μl DNA and 1 μl of each primer (10 pmol/μl stock). An initial denaturation step of 95°C for 3 min was applied, followed by 45 cycles of denaturation (95°C for 45 sec), annealing (49–54°C for 45, according to Table 3) and polymerization (68°C for 45 sec). An extension step of 10 min at 72°C was included after the end of the 45 cycles. Eighteen (18) μl were digested from each PCR product, in a total volume of 30 μl, following the instructions of the restriction enzyme supplier (Fermentas). Ten (10) μl of each PCR product were electrophoresed in 1.5% agarose gels, using 1X TAE buffer.

**Table 3:** PCRs for determining mitochondrial medfly haplotypes through PCR-RFLP tests, primer sequences and binding sites, size of expected amplicon and annealing temperatures, along with respective restriction enzymes are indicated.

Restriction enzyme	Primer pair	Primer position	PCR fragment (bp)	PCR Tm (°C)
nEcoRV-F	5’- ATTGACCCAGATACAGGAGCTT-3	8951–8972	741	54
nEcoRV-R	5’- GAGTATGTGAAGGTGCTTTAGGAC-3’	9668–9691		
Xba I—F	5’-TCCTAAACCATCTCACCC-3’	7821–7838	537	54
Xba I—R	5’-GTGGGGGAAATATTCGAGAG-3’	8337–8357		
MnlI(Cc7815)-F	5’- ACTAATCCTAAACCATCTCACCC-3’	7851–7874	766	56
MnlI(Cc8581)-R	5’- ACCTTCTATAACATTGTGGTGGT-3’	8581–8604		
Hae III—F	5’-GTATGTTTAATTCGACACTT-3’	5457–5476	532	49
Hae III—R	5’-CATTCATGGTATAGTCCAAT-3’	5969–5988		

## Results

### Cryopreservation of the medfly Vienna 8 GSS

Six attempts to cryopreserve medfly Vienna 8 GSS embryos using the previously published protocol [38] were not satisfactory mainly due to the low hatch rate (<2%) observed after the revival of the cryopreserved embryos. To solve this problem, optimization of the incubation time of the collected eggs at different temperatures to reach the right embryonic stage was conducted. The incubation at 29°C for 27 hours as previously recommended was repeatedly unsuccessful because the optimum developmental stage for the cryopreservation, which is characterized by the helical shape of the embryos gut with minimum amount of yolk, could not be identified (Fig 1A). Incubation of embryos for longer times (29–32 hours) was also tested but the recovery rate remained very low (< 2%). In addition, several challenges i.e. embryos clump formation and strong bacterial contamination during the recovery of the embryos in Schneider medium were frequently observed. The clumping issue was solved by spraying with water during the incubation in 10% ethylene glycol. It is worth mentioning that attempts to use antibiotics (Antibiotic-Antimycotic solution at final concentration of 1%, Gibco) in the recovery medium showed serious negative impact on the recovery rate. After the failed attempts at 29°C, incubation of embryos at lower temperatures, such as 21°C for 68 hours and 24°C for 48± 0.5 hours was tested. The later one turned to be successful as it resulted in to 38% hatch rate for the manually treated and robot handling embryos. In the first cryopreservation run (manual method) 351 larvae were hatched and produced 190 pupae which initiate the line V8-118. The second run was conducted using a home-made robot produced 458 hatched larvae developed to 284 pupae which initiate the line V8-228 (Fig 1B). As expected for the Vienna-8 strain a female deficiency was observed, evident in both pupae and adult stages. More specifically, 124 brown pupae (produced 113 males) and 66 white pupae (produced 58 females) initiated V8-118 line while 153 brown pupae (that produce 152 males) and 131 white pupae (produced 116 females) initiated the V8-228 line. It is important to note that the hatch rate for the dechorionated and permeabilized embryos controls (no treatment with liquid nitrogen) was 63.8% and 47.9% respectively.

### Quality control of the cryopreserved lines

Starting from 40 ml of pupae, V8-118 and V8-228 lines produced 1882 and 2008 adult flies, respectively. These numbers were comparable (although lower), with the V8-F40 used as a control (2203 adult flies) (Table 1). This difference is mainly attributed to the decreased number of females, since differences in the number of males are low (ranging from 1057 to 1265). However, as evident from the 5400 egg screening (see below), these differences in recovery rates at 25°C were not replicated. As expected, there was a deviation from the 1:1 sex ratio, which is consistent with the *tsl* character of the strains [15]. There are substantial differences among the strains regarding the sex ratio (V8-F40: 1.24; V8-118: 1.28; V8-228: 1.70) (Table 1), however, this was not replicated in the 5400 egg screening (see below plus Table 1) and requires further investigation. Importantly, no recombinants were observed (meaning no white pupae males or brown pupae females), consistent with the stability expected for the Vienna 8 strain [15] (Table 1).

**Fig 2:**
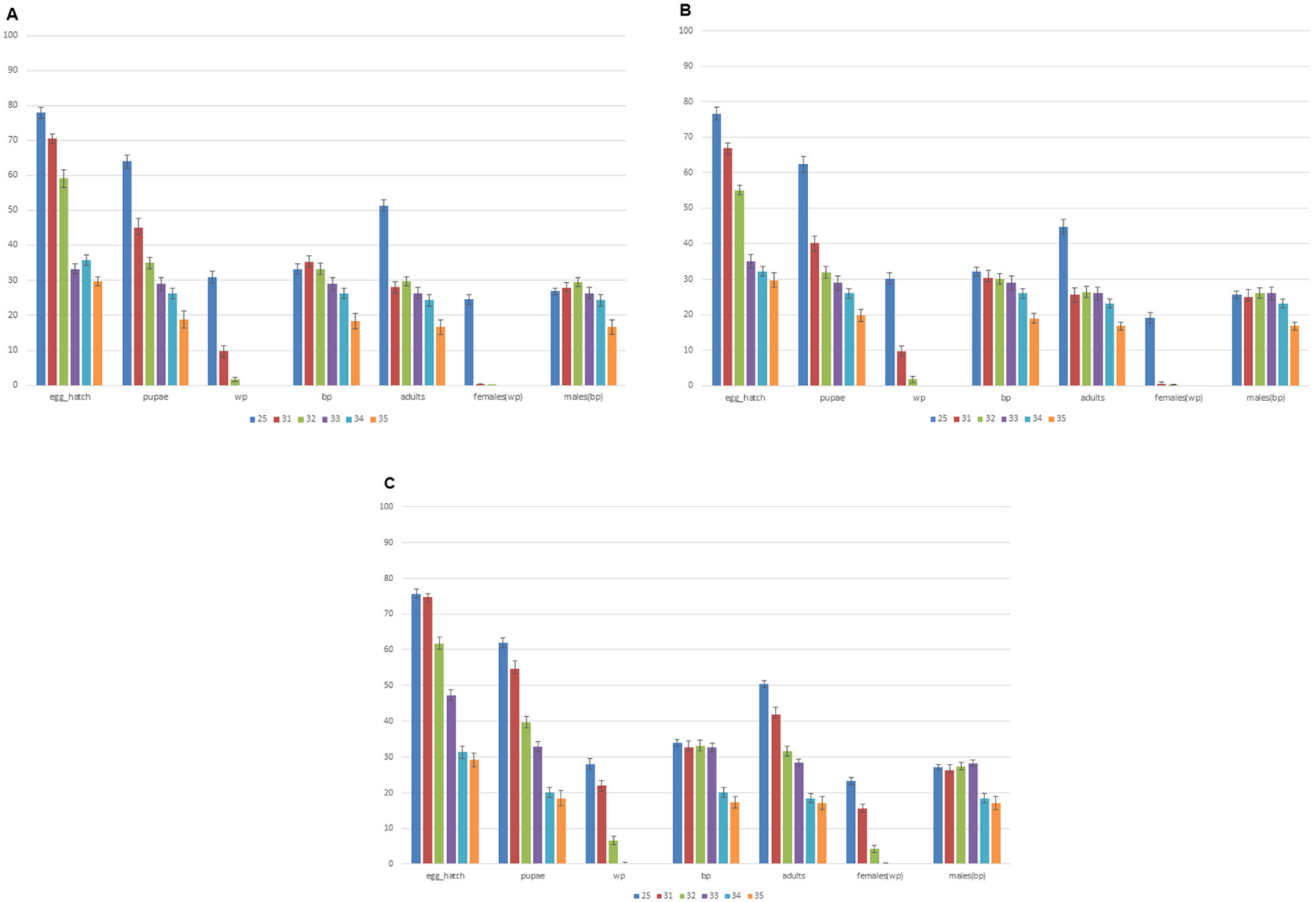
Percentages of egg hatch, pupation (total, white and brown pupae) and adult emergence (total, females and males) in different temperatures (25, 31, 32, 33, 34 and 35°C) according to the *tsl* screening described in Table 2. Results derived from a total of 5400 eggs QC screening (standard errors are indicated). (A) V8-118, (B) V8-228 and (C) V8-F40.

**Fig 3:**
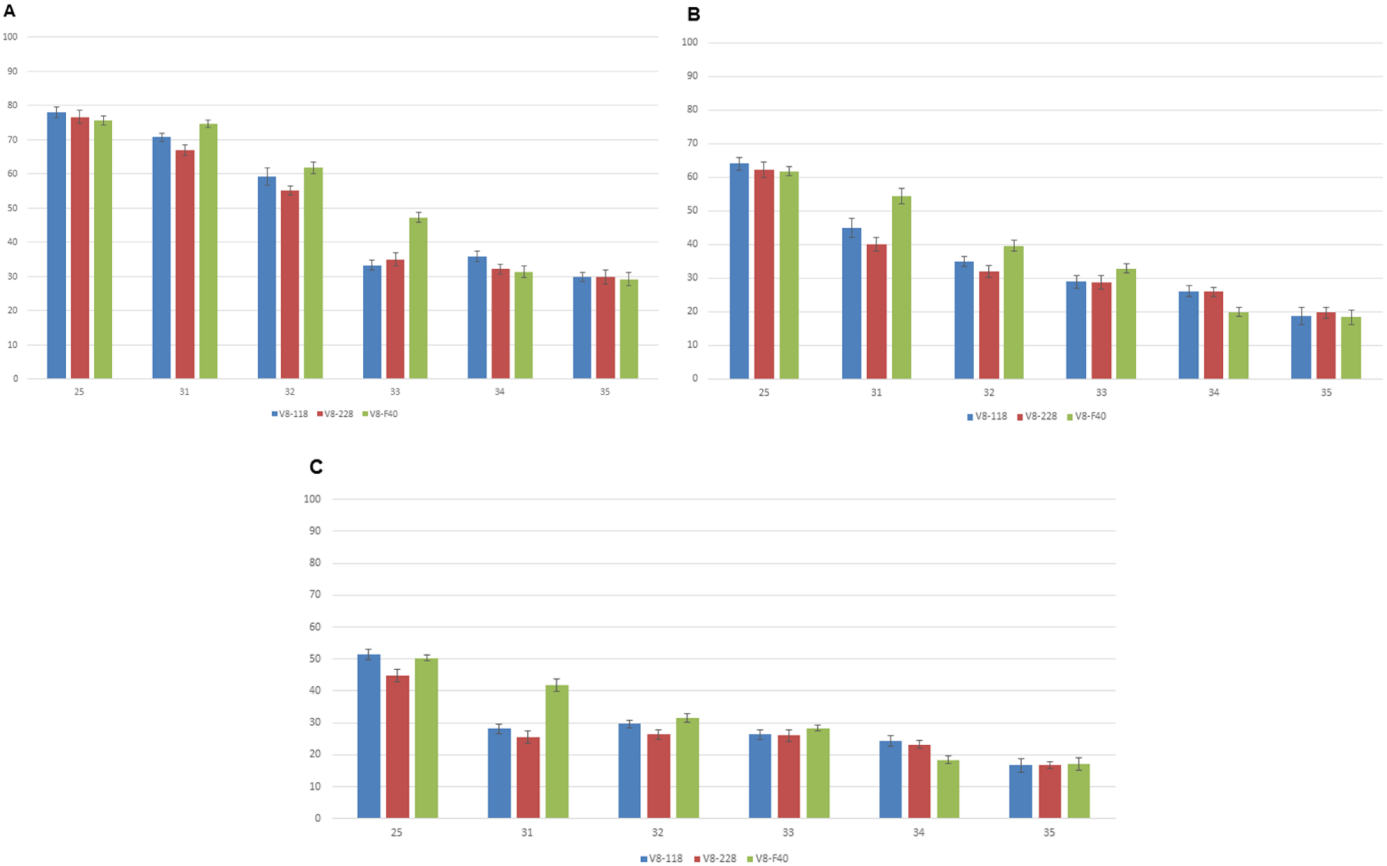
Comparison of the three strains (V8-118, V8-228 and V8-F40) in five different temperatures (25, 31, 32, 33, 34 and 35°C), according to the *tsl* screening described in Table 2 (standard errors are indicated). (A) egg hatch (per 100 eggs), (B) pupation rates (per 100 eggs) and (C) adult emergence (per 100eggs).

**Fig 4:**
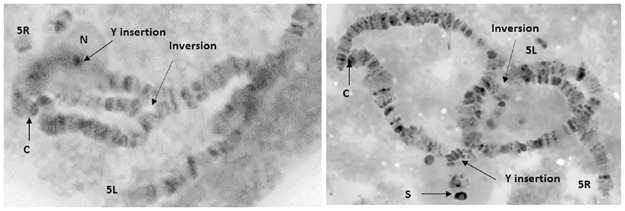
Polytene chromosomes derived from trichogen cells of 5–6 days old male pupae of the V8-118 line. The 5L and 5R chromosome arms are marked. The D53 inversion and the Y-autosomal translocation are indicated. N: nucleolus, C: centromere, S: dense sphere, representing the Y chromosome.

**Fig 5:**
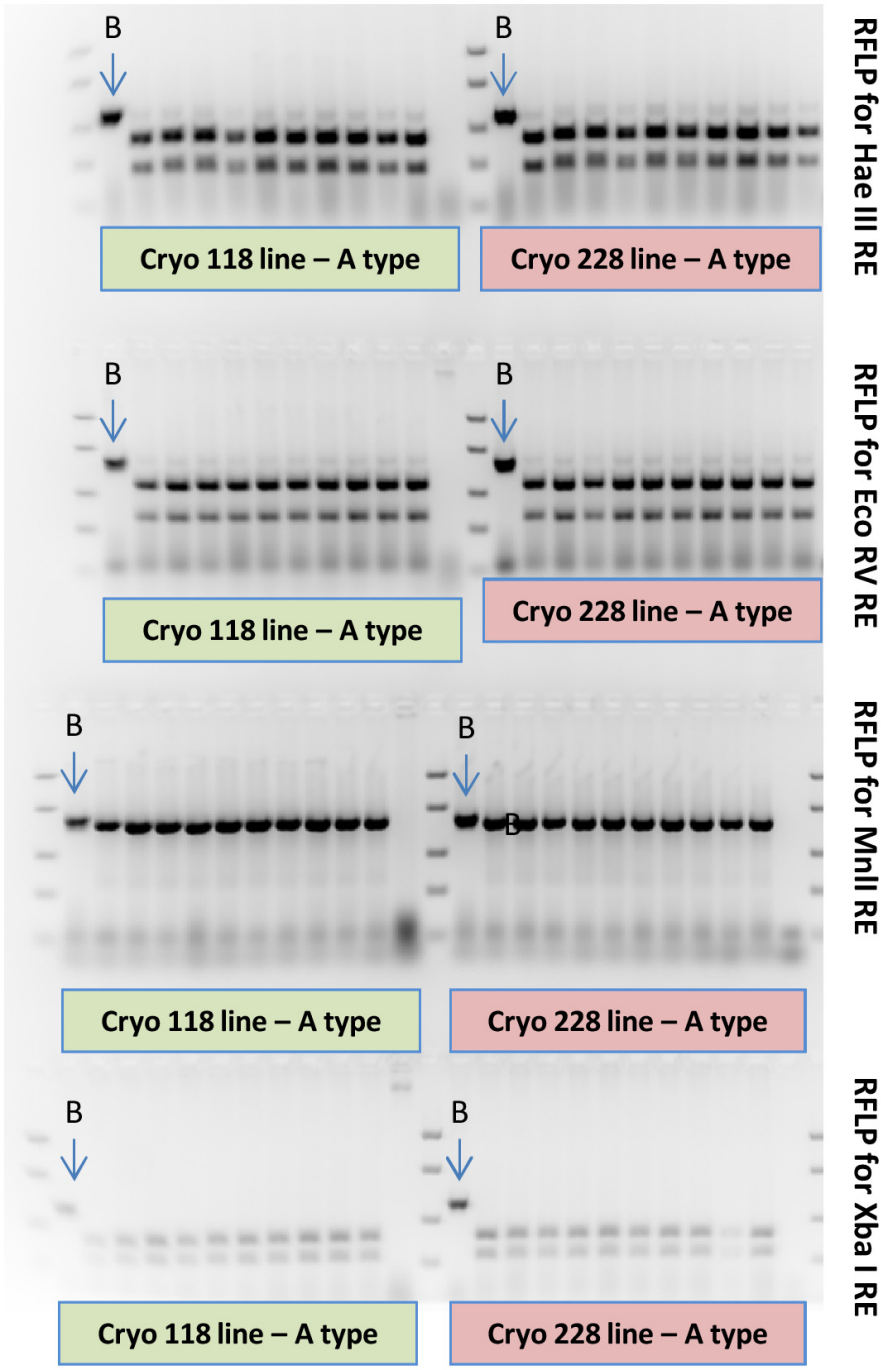
Test for the presence of RFLP markers identified for the original line before cryopreservation procedure. All flies derived from the Vienna 8 strain exhibit the expected AAAA haplotype. As a control, a fly belonging to the BBBB haplotype was included in the analysis (see 1st lane next to the marker). As a DNA marker, the FastRuler Low Range DNA Ladder was included in the electrophoresis.

A total of 5400 eggs were used per strain, as described in Table 2, to simultaneously measure recovery rates, recombination rates and *tsl* character stability. The results per strain are shown in Fig 2A–2C. No significant differences were observed among strains regarding important parameters such as egg hatch rate, pupation rates, adult emergence rates and stability of the *tsl* character (Fig 3A–3C). Egg hatch at 25°C was similar for the three tested strains, showing no statistically significant differences (V8-118 = 78%, V8-228 = 76,67%, V8-F40 = 75,67%; ANOVA: *P* = 0.604) (Fig 3A). Pupation rates (egg to pupae recovery) were also comparable for the three tested strains, showing no statistically significant differences (V8-118 = 64.11%, V8-228 = 62.33%, V8-F40 = 61.89%; ANOVA: *P* = 0.663) (Fig 3B). Regarding adult emergence, some differences were observed among the three tested strains; however, they were mainly attributed to females rather than males. More specifically, at 25°C, adult emergence ranged between 44.77% and 51.44% (V8-118 = 51.44%, V8-228 = 44.77%, V8-F40 = 50.33%). While recovery rates of males were similar for all three tested strains, ranging between 25.67% and 27.11% (V8-118 = 26.89%, V8-228 = 25.67%, V8-F40 = 27.11%), there were greater differences in female emergence, ranging between 19.11% and 24.55% (V8-118 = 24.55%, V8-228 = 19.11%, V8-F40 = 23.22%) (Fig 3C). However, these differences were not statistically important (ANOVA: *P* = 0.604). In general, variation was observed in the numbers of white pupae and respective numbers of females at 25–32°C, probably due to the interaction of the temperature with the *tsl* character. Nevertheless, these differences do not affect the main findings, which are consistent in all tested strains: a) complete absence of recombinants (either white pupae males or brown pupae females) and b) complete absence of escapers, meaning no white pupae and/or females were observed at 33, 34 and 35°C (Fig 2A–2C). Regarding the sex ratio, it was measured both as the ratio of white to brown pupae and as the ratio of females to males (Table 1 and Figs 3–5). As described also above, a generalized (small) deficiency of white pupae and subsequently, females, was observed in all tested strains at 25°C. Minor differences were observed among the strains, both regarding the white pupae to brown pupae ratio (V8-118 = 0.95, V8-228 = 0.95, V8-F40 = 0.84) and the males to females’ ratio (V8-118 = 1.08, V8-228 = 1.33, V8-F40 = 1.15) (Table 1 and Fig 2A–2C). However, none of these differences were statistically significant (ANOVA: *P* = 0.364 and *P* = 0.122, respectively).

### Cytogenetic characterization

Analysis of polytene chromosomes of the trichogen cells of 5–6 days old male pupae verified that both lines are stable, harboring both the Y—autosome translocation [T(Y;5)101] and the D53 pericentric inversion on chromosome 5 [In(5L-5R)50-59] (Fig 4). These results are the expected, based on the properties of the specific medfly Vienna 8 GSS used [15].

### Mitochondrial haplotypes

Ten flies per cryopreserved line (V8-118 and V8-228) were analyzed. After the PCR-RFLP test performed, all of them presented the expected mitochondrial haplotype (AAAA) (Fig 5) of the medfly Vienna 8 GSS, as described in [28].

## Discussion

The results of the current study provide the improved procedure to cryopreserve the medfly Vienna 8 GSS without affecting its genetic characteristics. Indeed, the cryopreservation didn’t have any major effect on any of the quality parameters assessed for the two cryopreserved lines established for this strain: egg to pupae and egg to adult recovery, lack of recombination and stability of the *tsl* character were as expected for the original Vienna 8 GSS. Cytogenetic analysis and analysis of mitochondrial haplotypes verified the chromosomal and genetic stability of the obtained lines. The fact that all experiments were performed in the 2^nd^ generation after the revival demonstrates that there is no need for extended periods of reviving and upscaling for the cryopreserved strains; this was achieved within two generations. The potential impact of the cryopreservation on the mating competitiveness of these two cryopreserved lines of the medfly Vienna 8 GSS as well as a cost-benefit analysis will be the focus of future studies.

Since its establishment in 2003, the medfly Vienna 8 GSS is world-wide used for the production of sterile males in at least ten mass rearing facilities, with the largest one (El Pino in Guatemala) producing up to 3 billion sterile males per week, for SIT applications in United States, Mexico and Guatemala [21]. Although the Vienna 8 strain has been proven to be very stable in terms of rearing and male mating competitiveness [47], the continuous use of any insect strain in large scale production facilities may result in to the production of sterile males with significantly reduced mating competitiveness [27, 29, 30, 48, 49]. It has been so far difficult to identify the causal factors underlying such deterioration phenomena, simply due to the absence of the possibility to compare between the reared strain and the original one, prior to its laboratory adaptation and domestication. Potential factors could be genetic changes associated with bottleneck, inbreeding or genetic drift [29]. Adaptation in artificial mass rearing diet consisting of preservatives and artificial oviposition substrates can improve rearing efficiency but may also affect the biological characteristics of the strain including its symbiotic community. Both genetic and “symbiotic” changes can be crucial for the loss of characters important [29], for SIT-based applications such as the male mating competitiveness. Additional factors which may result to the deterioration of a SIT strain could be strain contamination, human handling errors (where several strains are maintained), or the presence of pathogens and parasites [50]. The availability of the cryopreserved V-8 or other strains used in SIT action programs at the beginning of it domestication process or during first generations of mass rearing would allow comparisons with the same strain after several generations in mass-rearing. In this context, the cryopreservation technology—provided that it does not affect the strain- may restores the *status quo ante* and allows comparative analysis which could determine changes at the genetic level as well as at the symbiotic community that potentially be responsible for the strain deterioration [50]. Such comparison could also allow alerting if the strain deterioration problems can correlate with the domestication process of the strain or associated to other factors that are not associated with the age of the strain in mass rearing. Therefore cryopreservation can be additional tool for SIT manager to take the appropriate decisions.

The cryopreservation technology has been developed for many insect species such as *D*. *melanogaster*[51], *Lucilia cuprina* [33], *Musca domestica* [36], *C*. *hominivorax* [32], *Culicoides sonorensis* [52], *Anastrepha suspensa* [39], *Anastrepha ludens* [37], *Galleria mellonella* [53], *Pectinophora gossypiella* [54] and *Lucilia sericata* [34]. Cryopreservation technology for a wild type strain of medfly is also available [38]; but these protocols were not successful for the medfly Vienna 8 GSS due to the characteristics conferred by the strain having genetic markers induced for the construction of the sexing mechanisms. Our data suggest that this difference is most likely due to the presence of the thermal sensitive lethality (*tsl*) gene in the medfly Vienna 8 GSS which induced embryo lethality when incubated at temperatures higher than 25°C in the first 24 hours of development [15]. Therefore, once the temperature was adjusted from 29°C to 24°C for 48 hours, the cryopreservation protocol was successfully applied to the medfly Vienna 8 GSS.

The finding of this study is of utmost importance for SIT mass rearing facilities. This approach is also very important when developing or finding a strain with interesting genetic and biological features, and difficult to “replicate”, in order to preserve it before the accumulation of the possible detrimental effects associated with domestication and inbreeding or other factors. To further extend this, the usual practice of testing the competitiveness and efficiency of such strains against populations derived from the wild that are not available throughout the year and are used after a varying number of generations in the lab, would also benefit of the cryopreservation method. Taken together the results of this study confirm the usefulness of the cryopreservation technology and support its expansion and further implementation in insect pests and disease vectors of SIT importance.
